# “People will say that I am proud”: a qualitative study of barriers to bed net use away from home in four Ugandan districts

**DOI:** 10.1186/1475-2875-13-82

**Published:** 2014-03-06

**Authors:** April Monroe, Steven A Harvey, Yukyan Lam, Denis Muhangi, Dana Loll, Asaph Turinde Kabali, Rachel Weber

**Affiliations:** 1 Johns Hopkins Bloomberg School of Public Health, 615 N. Wolfe Street, Baltimore, MD 21205, USA; 2 Makerere University, School of Social Sciences, P.O. Box 7062, Kampala, Uganda; 3 Johns Hopkins Bloomberg School of Public Health Center for Communication Programs, 111 Market Place, Suite 310, Baltimore, MD 21202, USA; 4 Team Initiatives Ltd, Teachers House, Plot 28/30 Bombo Road, P.O. Box 3963, Kampala, Uganda

**Keywords:** Malaria, Insecticide-Treated Bednet, Sleeping outdoors, Barriers, Net use, Qualitative research, Funerals, Conflict, Uganda

## Abstract

**Background:**

Despite increased access and ownership, barriers to insecticide-treated bed net (ITN) use persist. While barriers within the home have been well documented, the challenges to net use when sleeping away from home remain relatively unexplored. This study examines common situations in which people sleep away from home and the barriers to ITN use in those situations.

**Methods:**

To explore these issues, a group of researchers conducted 28 in-depth interviews and four focus groups amongst adults from net-owning households in four Ugandan districts.

**Results:**

In addition to sleeping outside during hot season, participants identified social events, livelihood activities, and times of difficulty as circumstances in which large numbers of people sleep away from home. Associated challenges to ITN use included social barriers such as fear of appearing proud, logistical barriers such as not having a place to hang a net, and resource limitations such as not having an extra net with which to travel. Social disapproval emerged as an important barrier to ITN use in public settings.

**Conclusions:**

Unique barriers to ITN use exist when people spend the night away from home. It is essential to identify and address these barriers in order to reduce malaria exposure in such situations. For events like funerals or religious “crusades” where large numbers of people sleep away from home, alternative approaches, such as spatial repellents may be more appropriate than ITNs. Additional research is required to identify the acceptability and feasibility of alternative prevention strategies in situations where ITNs are unlikely to be effective.

## Background

The World Health Organization estimates there were 219 million cases of malaria in 2010, most in sub-Saharan Africa. Among African countries, Uganda ranks fifth in malaria cases and ninth in malaria-related deaths [1]. Insecticide-treated mosquito nets (ITNs) are widely recognized as an effective tool for reducing malaria-related morbidity and mortality [2]. Over the past decade, the Ugandan government has initiated large ITN distribution campaigns and intensified education on malaria prevention [3]. These efforts have contributed to nearly universal awareness of ITNs and increases in ITN coverage, including a fourfold increase in ITN ownership between 2006 and 2011 [4,5].

Nonetheless, barriers to consistent net use remain. Insufficient ITNs in a household, feeling hot or suffocated, perceived absence of mosquitoes, and to a lesser extent inconvenience are commonly reported obstacles in Uganda and elsewhere [6,7]. Although substantial research on such barriers exists, it has largely focused on net use in the home or when sleeping outside in the hot season. Situations in which people sleep away from home may also increase malaria risk and present a unique set of challenges. This article focuses on those situations and challenges.

## Methods

**Figure 1:**
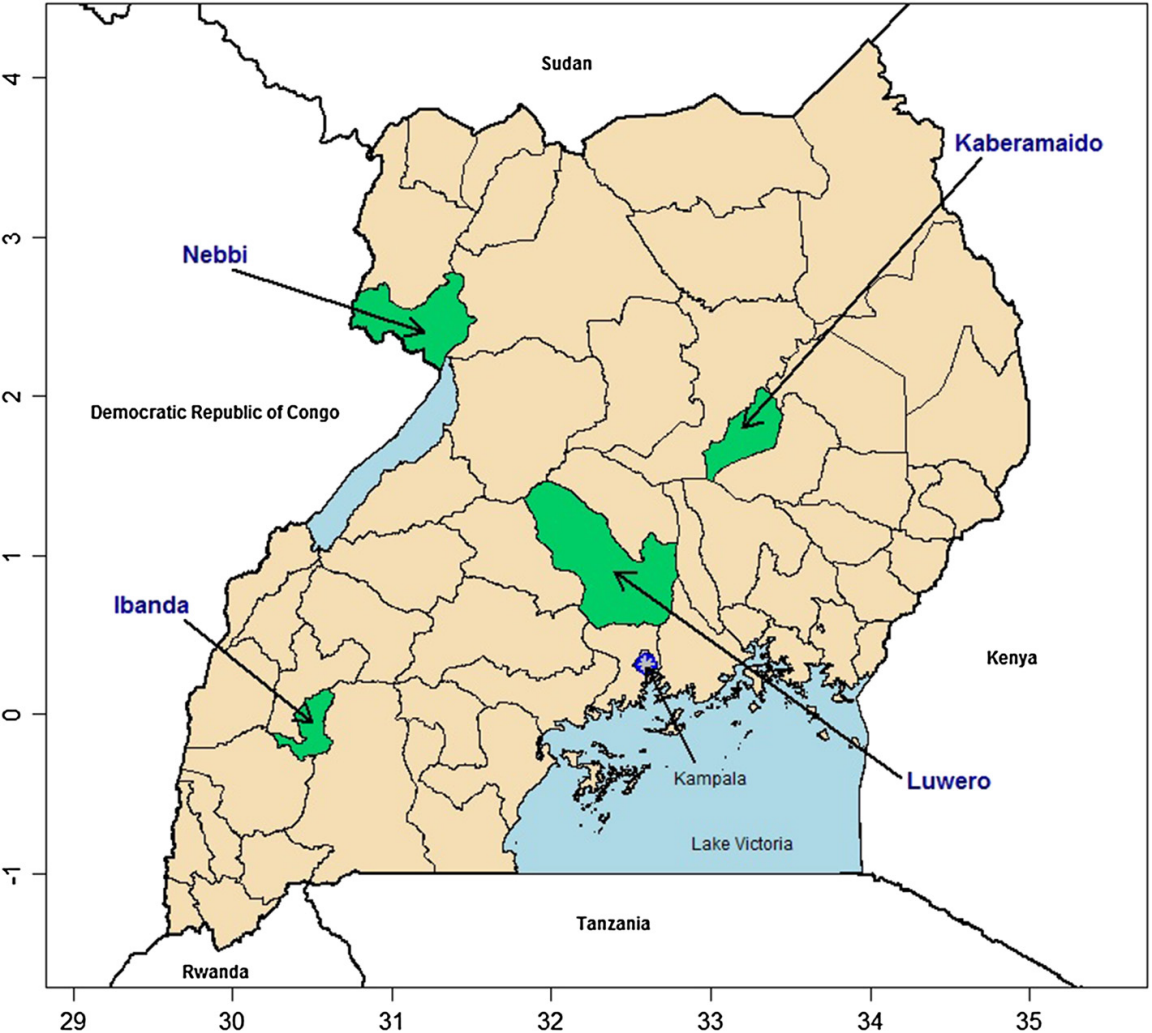
Map of Uganda with study districts (Ibanda, Kabermaido, Luwero and Nebbi) indicated.

This study occurred in four purposively selected districts in Uganda: Ibanda, Kaberamaido, Luwero, and Nebbi, representing the Western, Eastern, Central, and Northern Regions, respectively (Figure 1). Data collection took place in one rural and one peri-urban village within each district. It included in-depth interviews (IDIs), sleeping space questionnaires, and focus group discussions (FGDs). An initial round of data collection took place in Luwero and Nebbi in March 2012. A second round took place in all four districts in January 2013. This paper reports on the IDIs and FGDs from round two. Other results are reported elsewhere [8].

IDI and FGD participants were eligible for the study if they were eighteen or older and their compound owned at least one net. All participants provided oral informed consent. Village leaders assisted with selection of IDI households, which was done purposively to maximize geographic and socio-economic variation. The household head or his/her spouse was invited to participate.

Within selected households, an adult not chosen for the IDI could be recruited for a focus group. Additional focus group respondents were recruited by convenience sampling. One male and one female focus group each were conducted in Luwero and Nebbi during round one and in Ibanda and Kaberamaido during round two.

IDIs and FGDs were conducted in local languages, audio-recorded, transcribed and translated into English. Transcripts were coded in ATLAS.ti v. 6.2 [9]. An initial codebook was developed based on the research questions, as well as review of the transcripts. Ten transcripts were randomly selected and coded by two members of the research team. A final codebook was created based on themes identified during trial coding and team discussion. All transcripts were then coded using the final codebook. While initial codes for sleeping away from home were drawn from specific research questions, analysis of transcripts was largely inductive, with circumstances and barriers to net use emerging from the data.

Ethical approval for the research was secured from the Johns Hopkins University Bloomberg School of Public Health Institutional Review Board in Baltimore, Maryland, and from the Joint Clinical Research Center and the Uganda National Council for Science and Technology Institutional Review Boards in Uganda.

## Results

The results reported here are based on the 28 in-depth interviews and four focus groups conducted in January 2013. Each focus group included 10 participants. Table 1 shows the breakdown of both interview and focus group participants by gender and district.

**Table 1:** Interviews and focus groups by district

	**In-depth interviews**			**Focus group discussions**		
				**# FGDs (# participants)**		
**District**	**Males**	**Females**	**Total**	**Males**	**Females**	**Total**
Ibanda	2	4	6	1 (10)	1 (10)	2 (20)
Kaberamaido	0	6	6	1 (10)	1 (10)	2 (20)
Luwero	2	6	8	0	0	0
Nebbi	5	3	8	0	0	0
**Total**	**9**	**19**	**28**	**2 (20)**	**2 (20)**	**4 (40)**

### Reported reasons for spending the night away from home

Participants cited multiple reasons for spending the night away from home, including social events, activities related to livelihood, and what is referred to here as times of difficulty (Table 2).

**Table 2:** Reported reasons for spending the night away from home

**Social events**	**Livelihood activities**	**Times of difficulty**
• Funerals	• Occupational activities	• Domestic disputes
• Weddings	• Household-level chores	• Insurgency
• Religious events		• Threats of violence
• Celebrations		• Sickness
• Visiting friends and family		• Mental illness
• Discos		• Intoxication
		• Prison

### Social events

Men and women from all districts mentioned large community events as commonplace circumstances for spending the night away from home. As noted by a woman from Nebbi, such events might entail *“celebrations or sadness.”* Funerals were the most frequently cited event in general, while weddings were the most common celebration. Informants also mentioned parties and religious “*crusades*,” large religious gatherings that last between a day and a week.

A smaller number of participants mentioned going to discos and bars as social activities that might involve staying out all night. Male and female interviewees from all districts, but especially Ibanda, also cited smaller-scale social engagements, such as overnight visits to friends and family.

### Livelihood activities

Participants mentioned several occupations that require spending all or part of the night outdoors, including police officer, security guard, soldier, fisherman and brick-maker.

At the household level, participants described chores such as herding animals and getting up early to milk cows. They also mentioned collecting “white ants” (an edible insect of the termite family) that emerges from the ground late at night and serves as a food source for many Ugandans:

*“When you have your animals but no corral to keep them, you also stay outside to prevent the animals from going into people’s gardens. And also during the time for catching white ants you stay outdoors to keep the time. For example, there are those that start from 9:00 p.m. to 11:00 p.m.; then others start from 1:00 a.m. to 4:00 a.m.”* (Female, Kaberamaido)

### Times of difficulty

Informants listed both family- and community-level conflicts that can cause those affected to sleep away from home. For instance, a number of female respondents mentioned domestic violence or disputes.

*“When I fight with my husband and he chases me out of the house,”* one Kaberamaido woman explained, *“I will sleep outside that night, and sometimes there is nothing to sleep on because this is an unplanned-for situation. You can’t access a net.”*

Reports of sacrificial killings around Luwero have led some residents to spend nights away from home in fear. Respondents report hiding outside, as well as having men sleep outside to protect the community.

*“Maybe if there is some crisis, like nowadays when we hear that some people are being killed, people may move out of their houses for safety… We have been hearing it on the radio, but as for us who experienced war, we would hide for safety. We would move from the house to the bush. But it is all the same: You would move into the bush and experience hardship and risk yourself.”* (Female, Luwero)

In Luwero and Kaberamaido, respondents cited civil war and insurgency as reasons to sleep away from home. As one Kaberamaido woman recalled, *“[in] the time of Kony there was insecurity, so we had to sleep outside and not in our homes or houses.”* (Note: the informant is referring to Joseph Kony, head of the Lord’s Resistance Army or LRA, a rebel group that has been fighting the government of Uganda for more than two decades).

Beyond strife, informants identified sickness as another time of difficulty when one might spend the night at a health centre either for treatment or to care for a family member. Some participants noted that health facilities have no nets whatsoever or insufficient nets to cover family members of patients. A few participants described sleeping outside when ill to get fresh air or be closer to the latrine. Mental illness, intoxication, and imprisonment were also cited, though less frequently.

### Barriers to net use away from home

Challenges associated with net use away from home tended to fall into three categories: social barriers, logistical barriers, and resource limitations (Table 3).

**Table 3:** Barriers to bed net use outside the home

**Social barriers**	**Logistical barriers**	**Resource limitations**
• Appearing “proud” (or prideful)	• Crowding and congestion	• Leaving other family members unprotected
• Appearing disrespectful	• No place to hang net	
• Expectation of staying up all night	• Inconvenient to carry net	
• Inconveniencing others	• Restriction of movement	
	• Up and down all night	
	• No time to grab net	
	• Net makes person highly visible	

### Social barriers

Participants across all four districts frequently cited perceived social barriers to net use, most often with respect to funerals. Many participants reported that if one used a net at a funeral, others would look down on them for being prideful:

*“You cannot carry your net to a [funeral],”* a woman from Luwero explained, *“because people will speak a lot about you. They will say that you are showing off in such a place when people are in sorrow.”*

While social disapproval was most often cited in relation to funerals, participants also expressed apprehension about bringing nets to weddings and other celebrations:

*“People will say that you are full of pride: ‘you see, he carries a net when he is going to a wedding.’ Therefore, people will talk ill about you and emphasize how you are such a bad person who thinks that he/she is rich.”* (Male, Ibanda)

The same fear extended to more intimate social gatherings, such as visiting friends and family. Participants noted that bringing a net could seem disrespectful to the host and felt it was important to graciously accept the accommodations offered.

*“Sincerely, this thing of visiting other people and carrying your own net is very complicated. So if I come visiting you, should I also bring along my bed sheets? I think that when I go visiting, I am supposed to put up with the sleeping arrangements that you have made for me. Surely, if my host puts me in a room to sleep, shall I say that – ‘you see, I have to hang up my net since I cannot sleep without one?’ To be sincere… we would have carried our nets when we go visiting but the surrounding circumstances from other people affect us, and we end up not taking our nets… If you go with a net to visit someone, he or she will say that you are proud; you go with your mattresses and your nets. So you see that this thing is very difficult.”* (Female, Ibanda)

### Logistical barriers

Logistics were another reported barrier to net use at funerals, weddings, religious events, visiting, and travelling. Participants reported lacking a place to hang a net, the inconvenience of untying and carrying a net, and the difficulty of using a net properly at a crowded event.

*“You cannot take along a mosquito net to a wedding; where do you think you will hang it? Won’t you be sleeping on chairs? Tell me then, how you would hang your net on such a sleeping space?”* (Male, Ibanda)

The threat of fires at funerals was discussed as well. *“Fire is always lit at the funeral,”* a woman from Ibanda explained. *“Therefore, if you go with a net it may be burnt and you may also end up getting burnt and in this case it won’t help you.”*

Participants also mentioned logistical barriers with regard to livelihood activities, indicating that nets would prevent people from being able to move freely. Concerning threats of violence, participants indicated that one often does not have time to think about bringing a net, that it is cumbersome to carry and hang if one needs to flee quickly, and that it increases the visibility of someone who is trying to hide. Participants also mentioned courtesy as a type of logistical barrier. For example, some interviewees noted that they did not want to disturb those sleeping around them at crowded events:

*“You cannot hang and use a net during religious crusades because you are not going to use it to accommodate five or six people around you. So instead of stumbling and inconveniencing other people, you choose not to use a net.”* (Female, Ibanda)

In addition, during certain social events, it is common for people to stay awake all night. For example, during wedding celebrations*, “nets are not used because the people are very many, and at night after getting drunk they start dancing, and so there is no way a net can be used.”* (Female, Kaberamaido)

### Limited net access

Across all districts, participants of both genders said they could not take a net with them when spending the night away from home because that would mean leaving other family members without a net. This led to not having a net for various circumstances, including weddings, funerals, and visiting.

## Discussion

Vector control is a fundamental pillar of malaria control. In the absence of a vaccine that is highly effective, long-lasting, and practical to administer at the population level, vector control is key to any malaria reduction, elimination or eradication effort. To date, only two vector control technologies have proven effective on a mass scale: indoor-residual spraying (IRS) and insecticide-treated bed nets [2,10]. IRS depends upon endophagic (indoor feeding) and endophilic (indoor resting) mosquito behaviour. It is not a portable strategy. An ITN is theoretically portable, but can only be effective away from the owner’s home if he or she is able and willing to transport and use it.

Previous research has focused primarily on barriers to ITN use at home, where the most commonly cited reasons among nets owners for not using a net are heat, absence of mosquitoes, and inconvenience [7,11-13]. Only a few studies have addressed extradomiciliary use. For instance, discussion about ITN use at funerals goes back almost 20 years [11]. However, a 2011 review found that the relatively small number of studies available provide insufficient evidence to develop strategies for overcoming even home-based barriers [7].

This study contributes to the evidence base by describing a variety of circumstances in which people spend the night away from home. Each circumstance involves overlapping social, logistical, and resource barriers to ITN use. In some cases, findings build upon existing evidence. For example, structural barriers have long been identified as impeding net use [11,13]. Results of the present study reinforce these findings, suggesting that such barriers are heightened when sleeping away from home. Not having a place to hang a net and crowding and congestion were problems commonly cited by study participants.

### Social barriers

Moreover, this study highlights the unique challenges associated with net use in the public domain. Social barriers, such as norms and disapproval deterring net use were mentioned most frequently with regard to funerals, reflecting their highly public nature. Over the past several decades, burial societies or funeral associations have emerged in Uganda and other African countries to provide financial and social support to family members of the deceased [14-17]. Funerals now involve entire communities and are meant to offer dignity and respect to the dead [18]. By social convention, one aspect of that dignity and respect involves not appearing prideful by sleeping in an ITN at a time of mourning.

Religious crusades are another potential venue of increased malaria risk. Such gatherings are common among various religious groupings in Uganda. While less frequent than funerals, they can attract thousands of people and are held in large open-air settings, such as parks and stadiums. During crusades of a day or two, participants may not sleep at all; during longer crusades participants typically sleep outside, particularly if they are coming from some distance to attend.

### Times of difficulty

Frequent mention among study participants of strife, violence, and the need to flee into the bush reflects Uganda’s post-independence history of conflict [19-21]. Such conflict can produce tremendous short and long-term health consequences [22]. Malaria prevention has been identified as a key consideration in conflict and post-conflict settings [23]. While the particular circumstances described by this study’s informants may be unique to Uganda, armed conflict and insurgency are common in many malaria-endemic countries. It may seem obvious that those fleeing for their lives do not stop to pack their bed nets or spend time figuring out how to hang them while on the run. It is perhaps less obvious that the memory of such experiences has a continued impact even after the violence has ended. Results from this study suggest it is a phenomenon worth additional investigation.

The 2013 Northern Uganda Conflict Analysis, sponsored by the UK Department for International Development (DFID) and conducted by a consortium of civil society organizations, concludes that scars from the conflict in Northern Uganda persist and that large-scale violence could re-emerge:

*“Incomplete or inadequate transitional justice, reconciliation and return processes …leave people depressed, in conflict with each other, their neighbours and authorities, and with a sense that the war is still not completely ‘over’”*[24].

Factors such as land grabbing, corruption, and competition for natural resources also contribute to this threat. In many areas, clashes between communities and government officials, violent disputes over boundaries or resources, and sexual and gender-based violence continue [24-29]. While the full extent of these occurrences is not well documented, heightened media coverage may exacerbate fears of violence and have negative consequences for vector-control measures like ITNs.

A number of female participants reported domestic disputes as a reason for sleeping away from home. This may be understood within the larger context of domestic violence in Uganda, where 65.3% of married women ages 15–49 report experiencing some form of spousal violence, including physical, sexual, or emotional abuse, at some point in their marriage, and 58.1% report experiencing it within the past 12 months [5,30]. In one rural Uganda study, 70% of men and 90% of women stated that beating a wife or female partner is justifiable under some circumstances [31]. Worldwide, violence against women leads to physical, mental, sexual and reproductive health problems, and may increase vulnerability to HIV [32]. This study’s results suggest that domestic violence may also have implications for malaria control. Further research is needed to explore the potential link.

Resource limitations reported by study participants are also consistent with studies identifying net access as the key impediment to use [33,34]. Based on experience distributing hundreds of millions of nets in recent years, LLIN campaign coordinators have determined that one LLIN per 1.6 persons in a household is needed to achieve universal coverage [35]. This figure represents a delicate calculus that balances impact with limited donor funds. But that calculus does not provide the extra nets that would be required to protect both household members who spend the night away from home and those who remain behind.

Some research has been conducted into alternative forms of malaria prevention among populations where ITNs are inaccessible or problematic, including the use of insecticide-treated clothing and spatial repellents [36,37]. Spatial repellents – chemicals that prevent human-vector contact within a given area – represent an emerging malaria prevention technology that could be useful, particularly for large social gatherings [38]. Although effective in creating vector-free spaces and important for preventing malaria, they remain under-utilized [38-40]. Context-specific uses, such as those identified in this study, should be explored.

### Limitations

This study is qualitative and thus involves the usual caveats that come with small, non-representative samples. Still, the fact that these data were collected in four regions purposively selected to capture different geographic and socio-political characteristics makes it more likely that the phenomena participants describe occur well beyond their specific communities.

Perhaps the more important limitation is the paucity of epidemiological, entomological, and prevalence data related to the phenomena study informants describe. For instance, it seems reasonable to assume that many members of the population attend funerals and weddings, but little is known about frequency of attendance. This makes it difficult to estimate the increased malaria risk such events might entail. The potential risk of religious crusades is even harder to quantify: while attendance at any one crusade may range from several hundred individuals to many thousands, little is known about how often these events occur or how often a given individual might participate. The same is true for domestic violence, occupational exposure, care for family members interned at health facilities, and similar phenomena. It is possible that the cumulative effect of these exposures is quite significant or relatively limited, but more detailed research would help the malaria control community determine the value of targeting malaria prevention resources towards protecting those who sleep away from home for different reasons. More research could also help determine where it is practical to promote ITN use within a specific context and where other protection measures such as spatial repellants or insecticide-treated clothing might be more effective.

## Conclusions

People sleep away from home for a variety of reasons, producing multifaceted barriers to bed net use. This study has identified behaviors rooted in Ugandan history and tradition that could have important implications for malaria transmission. While bed nets are a valuable malaria prevention tool, they are not a cure-all, but rather a piece of a larger prevention strategy. Future research should examine (1) in what circumstances it is and is not feasible to promote ITN use as a primary prevention method for those sleeping outside the home; (2) where ITN promotion *is* feasible, what adaptations are needed in promotion strategies for particular contexts; and (3) where ITN promotion is *not* feasible, what alternative options might be available now or in the near future.

## Competing interests

The authors declared that they have no competing interests.

## Authors’ contributions

AM was responsible for data analysis and interpretation, drafting the manuscript, and managing edits to the manuscript. SH provided oversight and input throughout data collection and analysis. SH also made substantial contributions to developing and revising the manuscript. YL aided in data analysis and provided substantial feedback and revision of the manuscript. DM oversaw data collection, provided contextual information, and provided extensive feedback on the manuscript. DL assisted with study design, oversaw data collection and provided feedback on the manuscript. AK oversaw data collection, and provided feedback on the manuscript. RW conceived of the original study design and provided feedback on the manuscript. All authors reviewed and approved the final manuscript.
